# RNA-Targeting Techniques: A Comparative Analysis of Modern Approaches for RNA Manipulation in Cancer Research and Therapeutics

**DOI:** 10.3390/genes16101168

**Published:** 2025-10-02

**Authors:** Michaela A. Boti, Marios A. Diamantopoulos, Andreas Scorilas

**Affiliations:** Department of Biochemistry and Molecular Biology, Faculty of Biology, National and Kapodistrian University of Athens, Panepistimiopolis, 15701 Athens, Greece; miboti@biol.uoa.gr (M.A.B.); imdiamantop@biol.uoa.gr (M.A.D.)

**Keywords:** RNA-targeting technologies, antisense oligonucleotides, RNA interference, CRISPR-Cas13 systems, RNA manipulation, post-transcriptional regulation, clinical utility, RNA-based treatments, aptamers, cancer research

## Abstract

RNA-targeting techniques have emerged as powerful tools in cancer research and therapeutics, offering precise and programmable control over gene expression at the post-transcriptional level. Once viewed as passive intermediates in the central dogma, RNA molecules are now recognized as dynamic regulators of cellular function, capable of influencing transcription, translation, and epigenetic regulation. Advances in high-throughput sequencing technologies, transcriptomics, and structural RNA biology have uncovered a diverse landscape of coding and non-coding RNAs involved in oncogenesis, drug resistance, and tumor progression. In response, several RNA-targeting strategies have been developed to modulate these transcripts, including antisense oligonucleotides (ASOs), RNA interference (RNAi), CRISPR-Cas13 systems, small molecules, and aptamers. This review provides a comparative analysis of these technologies, highlighting their molecular mechanisms, therapeutic potential, and current limitations. Emphasis is placed on the translational progress of RNA-targeting agents, including recent FDA approvals and ongoing clinical trials for cancer indications. Through a critical comparison of these strategies, this review underscores the growing significance of RNA-targeting technologies as a foundation for next-generation cancer therapeutics and precision oncology.

## 1. Introduction

For decades, the central dogma of molecular biology—describing the flow of genetic information from DNA to RNA to protein—has served as the foundation for understanding a variety of cellular processes and for replicating the related mechanisms *in vitro* [1,2,3]. RNA was long considered a short-lived molecule whose primary function was to act as messenger RNA (mRNA), produced during DNA transcription and subsequently translated into polypeptides by ribosomes [4,5,6]. However, advances in transcriptomics, as well as transcriptome analysis methods and technologies, have made clear that RNA molecules are not just passive intermediates of the gene expression process [7].

One of the most significant breakthroughs in RNA biology has been the development of high-throughput sequencing technologies, particularly RNA sequencing (RNA-seq) [8,9,10]. This approach enabled the comprehensive, unbiased, and quantitative analysis of the transcriptome with unprecedented depth and precision. Compared to earlier hybridization-based techniques, RNA-seq allows for the detection of novel transcripts, alternative splicing events, RNA modifications [11,12], and numerous non-coding RNAs (ncRNAs) [13], revealing that the majority of the human genome is transcribed into RNA, much of which carries regulatory functions rather than encoding proteins [7,14]. Additionally, the emergence of single-cell RNA sequencing has further advanced our understanding by revealing cell-to-cell variability and transcriptomic heterogeneity across tissues, developmental stages, and disease states [15,16]. These technological breakthroughs gave insights into RNA research, broadening the recognized roles of RNA well beyond protein translation, and changing our perception regarding RNA and its functions.

Beyond acting as template for protein synthesis, RNA plays diverse and essential roles within the cell. These molecules are key regulators of gene expression at both transcriptional and post-transcriptional levels, controlling a wide range of processes including chromatin remodeling, transcriptional activation or repression, mRNA splicing, editing, transport, localization, stability, and translation [17,18,19,20,21,22,23,24]. In addition to regulatory roles, some RNAs catalyze biochemical reactions, acting as ribozymes, while others serve as structural and functional components of large ribonucleoprotein complexes like the spliceosome and ribosome [25,26]. The functional diversity of the transcriptome is reflected by the various classes of ncRNAs, including microRNAs (miRNAs), small interfering RNAs (siRNAs), long non-coding RNAs (lncRNAs), and circular RNAs (circRNAs), as well as catalytically active RNAs [27,28]. These molecules play crucial roles in maintaining cellular homeostasis [29,30,31], while dysregulation of their biogenesis, processing, or activity has been implicated in various diseases, particularly in cancer [32,33,34,35,36,37]. More precisely, it is well-established that ncRNAs can promote tumor growth, invasion, and resistance to therapy or act as tumor suppressors. Due to their specific expression patterns and regulatory roles, they are increasingly recognized as promising therapeutic targets, as well as valuable tools in both basic research and therapeutics.

Despite the introduction of the discussed high-throughput approaches, functional characterization of RNA molecules has required the development of complementary technologies capable of targeting and manipulating RNA directly [38,39,40]. RNA-targeting technologies offer the ability to modulate gene expression post-transcriptionally, often with greater specificity and reversibility than DNA-targeting methods [41,42]. The growing recognition of RNA’s diverse roles in gene regulation, catalysis, and structural organization has further catalyzed the development of these technologies for both basic research and clinical applications. They have enabled the in-depth investigation of RNA functions, interactions, and localization, and they serve as powerful tools for studying the effects of RNA dysregulation, a hallmark of cancer [43,44]. The introduction of these approaches has transformed our ability to manipulate RNA molecules, offering new insights into cellular biology and enabling the development of innovative therapeutic strategies [45,46].

Several distinct RNA-targeting strategies have been developed, each exploiting unique molecular mechanisms to achieve post-transcriptional modulation. Among the most well-established are antisense oligonucleotides (ASOs) and RNA interference (RNAi), which utilize sequence complementarity to inhibit translation, affect splicing, or induce transcript degradation [47,48,49]. These approaches have already been translated into clinical settings and are used in FDA-approved therapies for genetic and neurological disorders. More recent innovations include CRISPR-Cas13 systems, which enable programmable RNA cleavage and editing, offering high specificity and modularity for both research and therapeutic applications [41,50]. Additionally, small molecules that bind structured RNA motifs have emerged as a strategy to alter RNA stability or function, especially for ncRNAs or pathogenic repeat expansions [51].

Despite these advances, several significant challenges remain in the field. The structural complexity and dynamic expression patterns of RNA, along with the difficulty of efficient intracellular delivery, continue to hinder the widespread application of RNA-targeting technologies [52,53]. Additional obstacles include off-target effects, unintended immune activation, and variable efficacy depending on cellular or disease context [52,53,54]. Moreover, many current strategies are constrained to specific cell types or pathological conditions, highlighting the need for continued innovation to improve specificity, potency, and delivery mechanisms. Nonetheless, the ability to directly and precisely manipulate RNA holds great potential for studying gene function, dissecting disease mechanisms, and developing next-generation therapeutics with unprecedented precision.

This review examines the expanding toolkit of RNA-targeting technologies—from antisense oligonucleotides and RNA interference to CRISPR-Cas13 and RNA-binding molecules—and their growing relevance in medical oncology. We focus on their underlying molecular mechanisms, preclinical and clinical progress, therapeutic applications, and the specific opportunities they present for treating complex diseases such as cancer. We also address the key challenges that continue to limit their widespread clinical adoption and discuss their potential advances for overcoming these limitations. Through a critical analysis of recent developments, this review aims to highlight the current impact and the future potential of RNA-targeted approaches in cancer research and therapeutics.

## 2. Diverse Strategies for Post-Transcriptional RNA Modulation

RNA-targeting technologies have become essential tools in cancer research, offering precise and versatile modulation of gene expression at the post-transcriptional level [55]. This section provides a comparative overview of both established and emerging RNA-targeting strategies that have been developed and optimized to investigate RNA functions and regulatory mechanisms in cancer biology. Traditional methods such as antisense oligonucleotides (ASOs) and RNA interference (RNAi) are discussed alongside more recent innovations like the CRISPR-Cas13 system and aptamers, which have significantly expanded the toolkit for transcriptome manipulation. The present section focuses on the molecular mechanisms underlying each approach, their optimization for specificity and efficiency, and their functional applications in cancer research. In addition, we discuss the fundamental distinctions among these strategies, highlighting their respective advantages and limitations in experimental processes.

### 2.1. Antisense Oligonucleotides: Sequence-Specific RNA Targeting

**Figure 1:**
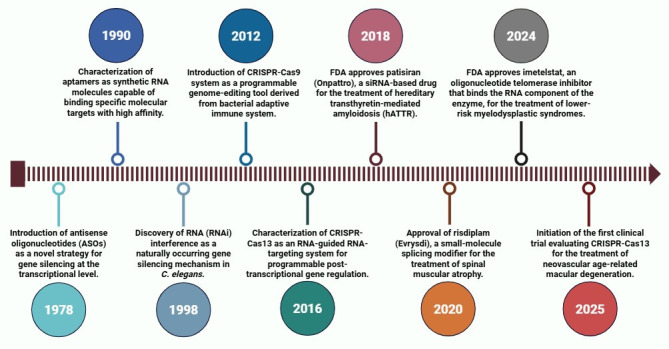
Key milestones in the development and application of RNA-targeting approaches. This figure summarizes major scientific and clinical milestones in the advancement of RNA-targeting technologies, spanning from the introduction of antisense oligonucleotides (ASOs) in 1978 to the initiation of the first clinical trial involving CRISPR-Cas13 in 2025. It presents significant discoveries in RNA biology and key regulatory approvals that underscore the growing potential of RNA-targeting strategies in cancer therapy.

One of the earliest methods developed for RNA manipulation is antisense oligonucleotides (ASOs) technology. First proposed in the late 1970s (Figure 1) [56], this approach involved the design of short, synthetic strands of DNA or RNA that can bind to a target RNA in a sequence-specific manner via Watson–Crick base pairing, enabling selective modulation of gene expression [57]. Over the decades, chemical modifications have been introduced to enhance ASOs’ stability, binding affinity, and cellular uptake, making them powerful tools for both basic research and therapeutic applications, especially in oncology.

Based on the type of chemical modifications they have been subjected to, ASOs are generally classified into three generations. First-generation chemistries include the widely used phosphate backbone modifications, e.g., phosphorothioate (PS). These modifications confer resistance to endonucleases and improve bioavailability but reduce the affinity for the target RNA [58]. Second-generation ASOs introduce modifications to the ribose sugar, particularly at the 2′-O position in RNA or the 2′ position in DNA. The most common examples of this generation are 2′-O-methyl (2′-OMe), 2′-O-methoxy-ethyl (2′-MOE) and 2′-fluoro (2′-F). These modifications enhance RNA binding affinity and further improve resistance to nuclease degradation [59]. Additionally, conformationally constrained DNA analogues like locked nucleic acid (LNA) and tricyclo-DNA (tcDNA) exhibit even greater binding affinity. LNAs contain a methyl bridge connecting the 2′-O and 4′ carbon atoms of the ribose ring [60], while tcDNAs introduce an ethylene bridge with a cyclopropane ring between the 3′ and 5′ carbon positions [61]. The presence of a chemical bridge locks the ribose ring in a favorable conformation for RNA hybridization, thereby enhancing binding efficiency. Third-generation chemistries involve more extensive modifications to the nucleobase or sugar-phosphate backbone. Typical examples include phosphonodiamidate morpholino oligomers (PMOs) and peptide nucleic acids (PNAs) [62,63]. Both PMOs and PNAs are uncharged, highly resistant to nucleases, and exhibit variable binding affinity to target RNA sequences, providing unique pharmacological properties ideal for therapeutic applications. [62,64].

#### 2.1.1. Dual Strategies: Degradation and Steric Blockade by ASOs

**Figure 2:**
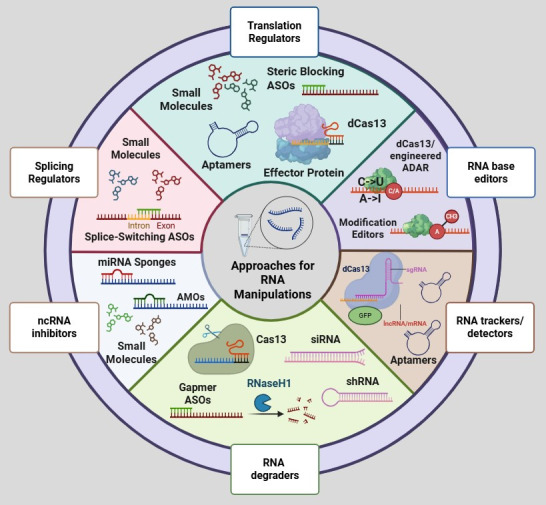
Applications for RNA-targeting technologies. A schematic diagram summarizing the major categories of RNA-targeting strategies and their respective applications. These include RNA degraders (e.g., siRNA/shRNA, ASOs, Cas13d) for transcript knockdown, RNA trackers and detectors for RNA detection or localization, RNA base editors for modulating RNA base modifications, splicing regulators for modulating alternative splicing events, translation regulators to control protein synthesis, and non-coding RNA (ncRNA) inhibitors targeting oncogenic or regulatory RNAs.

Based on their mechanism of action, ASOs are categorized into two main functional classes: gapmer ASOs, which induce degradation of target RNA transcripts, and steric-blocking ASOs (SBOs), which bind to their target RNA and sterically hinder its interaction with RNA-binding proteins or components of the cellular machinery (Figure 2) [65]. Both approaches enable precise modulation of gene expression at post-transcriptional level, but through distinct molecular pathways.

**Figure 3:**
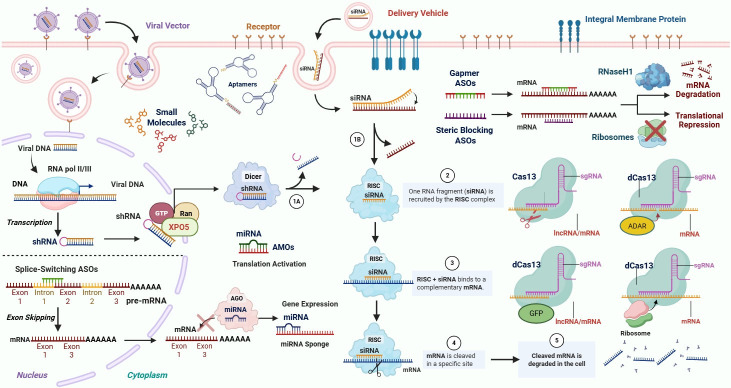
Mechanisms of RNA-targeting systems for RNA manipulation. This figure illustrates the molecular mechanisms of major RNA-targeting platforms used in cancer research and therapeutics. It includes antisense oligonucleotides (ASOs) subclasses, which act through RNase H1-mediated degradation or modulation of pre-mRNA splicing and protein interactions. RNA interference (RNAi) mechanisms via siRNA (1A→5) and shRNA (1B→5) are depicted, highlighting Dicer processing, RISC incorporation, and mRNA cleavage. CRISPR-Cas13 and catalytically inactive dCas13 systems are shown as programmable tools for targeted RNA cleavage, transcript editing, or RNA localization.

Gapmer ASOs are chimeric oligonucleotides designed to induce RNA degradation via RNase H1-mediated cleavage (Figure 3) [66,67]. Structurally, they consist of a central region of deoxyribonucleotides—the “gap”—flanked by chemically modified ribonucleotides on both the 5′ and 3′ ends [68]. These modifications enhance target binding affinity and prevent recognition by RNase H1, increasing nuclease resistance. Upon sequence-specific binding to the target RNA, the central DNA region forms a DNA/RNA duplex that is recognized by RNase H1, an endogenous endonuclease that selectively cleaves the RNA strand of the hybrid duplex while leaving the ASO intact for subsequent target recognition and cleavage [69]. This mechanism results in degradation of the target transcript and has been extensively applied in cancer research to downregulate oncogenic transcripts, disrupt tumor survival pathways, and overcome drug resistance. The incorporation of nuclease-resistant flanks improves pharmacokinetic stability and minimizes off-target interactions, while the DNA “gap” maintains the functional compatibility required for efficient RNase H1 recruitment and catalytic activity.

In contrast, SBOs exert their function by directly binding to specific sequences within target RNA molecules without inducing degradation (Figure 3) [65]. Unlike gapmer ASOs, which recruit RNase H1 to cleave the target RNA, SBOs act through a physical, occupancy-based mechanism. Upon binding, the oligonucleotide sterically hinders the access of RNA-binding proteins, components of the spliceosome or ribosome, and other elements of the RNA-processing machinery [70,71]. This interference can disrupt essential interactions involved in RNA splicing, translation initiation, or RNA stability, thereby modulating the fate and function of the RNA transcript. By selectively targeting *cis*-acting regulatory elements, SBOs can redirect alternative splicing events, alter mRNA maturation, or prevent the recruitment of translational machinery [57]. A notable subclass of steric-blocking ASOs is the splice-switching ASOs (ssASOs), which specifically bind to pre-mRNA splice sites and modulate splicing [68,72].

In addition to modulating mRNA processing, SBOs have been effectively used to target ncRNAs, particularly miRNAs. Anti-miRNAs (AMOs) are a class of SBOs designed to bind mature miRNAs, preventing their interaction with target mRNAs and thereby inhibiting miRNA-mediated repression. Through these diverse, non-degradative mechanisms, SBOs enable precise post-transcriptional regulation of gene expression while preserving RNA integrity, making them valuable therapeutic tools for correcting splicing defects, modulating protein expression, and inhibiting oncogenic miRNA activity.

#### 2.1.2. ssASOs: Splicing Modulation Using Steric-Blocking ASOs

In contrast to the gapmer-based mechanism that results in transcript degradation, ssASOs modulate pre-mRNA processing without inducing RNA cleavage (Figure 3) [73]. These oligonucleotides bind to specific splice sites or *cis*-regulatory elements like exonic splicing enhancers (ESEs), silencers (ESSs), or intronic elements, and direct alternative splicing [74]. Depending on the target site and the chemistry of the oligonucleotide, ssASOs can induce exon skipping or exon inclusion, thereby restoring or modifying the coding potential of the mature mRNA. ssASOs are commonly used to restore or disrupt reading frames, leading to increased protein levels for loss-of-function (LoF) variants or decreased protein levels for toxic gain-of-function (GoF) variants, respectively, dysregulations that are commonly observed in cancer [75,76,77]. ssASOs are typically composed of fully modified nucleotides, such as PMOs or LNAs, which are resistant to RNase H activity. This ensures the integrity of the RNA while allowing selective modulation of splicing events [78,79].

AMOs are synthetic ASOs designed to bind with great specificity to mature miRNAs (Figure 3) [80]. Once bound, they sterically hinder the miRNA from interacting with its mRNA targets and from being loaded into RISC, effectively neutralizing its gene-silencing function [81]. Mechanistically, AMOs resemble the action of gapmer ASOs but differ from them, since they typically do not induce degradation of their targets; instead, they function through competitive inhibition of miRNA–mRNA interactions [81,82,83].

Much like ASOs, AMOs are often subjected to chemical modification such as 2′-OMe, LNAs, or PS backbones to enhance nuclease resistance, binding affinity, and cellular uptake. These modifications improve the stability and efficacy of AMOs *in vivo* [83]. However, due to the transient nature of AMOs, inhibition of miRNA function is short-lived, necessitating multiple administrations to achieve a long-term knockdown effect.

### 2.2. Beyond AMOs: Using miRNA Sponges for miRNA Modulation

In cancer, numerous miRNAs are dysregulated, with oncogenic miRNAs (oncomiRs) often being upregulated. The overexpression of these miRNAs leads to the post-transcriptional repression of tumor suppressor genes, thereby promoting cancer development, proliferation, and metastasis [84]. While AMOs have been widely used to counteract these effects, additional RNA-based strategies such as miRNA sponges have also been developed. In cancer research, AMOs and sponges have been extensively used to investigate the functional relevance of well-characterized oncomiRs such as miR-21, miR-155, and the miR-221/222 cluster, which are frequently overexpressed across multiple tumor types. Functional inhibition of these miRNAs has revealed their critical roles in regulating apoptosis, cell cycle progression, metastasis, and immune modulation, underscoring their therapeutic potential and importance in cancer biology [85,86,87].

#### miRNA Sponges: Decoy Transcripts for Sustained miRNA Inhibition

In contrast to synthetic AMOs, miRNA sponges are typically DNA-encoded RNA transcripts engineered to contain multiple tandem binding sites complementary to one or more miRNAs [83,88]. These binding sites are often designed to match the miRNA seed region—critical for target recognition—allowing the sponge to sequester not just a single miRNA, but entire families sharing the same seed sequence. By competing with endogenous mRNA targets, sponges reduce the availability of free miRNAs for RISC loading and activity [83,89].

miRNA sponges are usually delivered via plasmid or viral vectors, resulting in their stable expression within the cell [89]. This enables long-term inhibition of miRNA function, making sponges particularly suited for *in vivo* cancer studies. Compared to AMOs, sponges offer prolonged miRNA suppression but often exhibit lower specificity and may cause off-target effects due to broader miRNA family inhibition. Moreover, their efficacy can vary depending on promoter strength, high endogenous miRNA concentration, and cell type [83,90].

### 2.3. RNA Interference: Harnessing the Cell’s Machinery for Precise RNA Degradation

RNA interference (RNAi) is a naturally occurring, sequence-specific post-transcriptional gene silencing mechanism, first described in *Caenorhabditis elegans* (*C. elegans*) in 1998 (Figure 1) [91]. It is mediated by small double-stranded RNA (dsRNA) molecules that direct intracellular machinery to selectively degrade complementary mRNA targets, thereby preventing their translation into protein [92]. Since its discovery, RNAi has become an indispensable tool in functional genomics and a promising therapeutic strategy, especially in cancer where aberrant gene expression drives tumor growth and progression [93].

The primary effectors of RNAi-based approaches are small interfering RNAs (siRNAs) and short hairpin RNAs (shRNAs) [94,95]. More precisely, siRNAs are synthetic RNA duplexes, typically 21–23 base-pairs in length, with characteristic 2 nucleotides 3′ overhangs that allow them to be recognized by the enzymatic machinery of RNAi. These molecules are designed to be fully complementary to their target RNA, enabling precise, sequence-specific silencing [95]. To enhance the efficacy of RNAi-based molecules, siRNAs are chemically modified similarly to ASOs, most commonly with 2’-OMe, PS, and 2’-F. These modifications improve nuclease resistance, immune evasion and target specificity, making them more suitable for *in vivo* approaches and clinical applications [96]. In contrast, shRNAs are generated intracellularly through the transcription of DNA vectors (e.g., plasmids or viral systems) that have been previously transfected into the cell [94,97]. The produced shRNAs are folding and form stem-loop structures. After being exported to the cytoplasm, these structures are processed by the RNase III enzyme Dicer into siRNA-like duplexes [98].

#### 2.3.1. Mechanistic Insights into RNAi: How siRNAs and shRNAs Achieve Targeted Gene Silencing

RNAi-mediated gene silencing occurs through a multistep process involving recognition, loading, and cleavage of target transcripts. For synthetic siRNAs, the duplex is introduced into the cytoplasm where it is directly loaded into the RNA-induced silencing complex (RISC) (Figure 3). The sense (passenger) strand of siRNA is degraded, and the antisense (guide) strand directs RISC to complementary mRNA transcripts [99]. Subsequently, Argonaute 2 (AGO2) mediates precise cleavage between the 10th and 11th nucleotides relative to the guide strand’s 5′ end [100], resulting in rapid degradation of the transcript by exonucleases [99].

In the case of shRNAs, the RNAi pathway begins in the nucleus, where the hairpin-structured RNA is generated via transcription and exported to the cytoplasm by Exportin-5 [101], a protein that is not required for the activity of synthetic siRNAs. Once in the cytoplasm, Dicer processes the hairpin into a siRNA-like duplex, which enters the same downstream pathway as exogenous siRNA (Figure 3) [97]. This approach offers a more sustained knockdown due to the continuous intracellular production of the shRNA; however, it may also lead to acute cytotoxicity, particularly at high expression levels or with certain delivery systems.

#### 2.3.2. Limitations of siRNA and shRNA: Choosing the Optimal RNAi-Based Tool

While both siRNAs and shRNAs harness the cell’s endogenous RNA interference machinery to silence gene expression, they differ significantly in design, delivery, and duration of effect (Table 1). siRNAs are synthetic molecules delivered directly into the cytoplasm, offering rapid and transient silencing with minimal cellular processing requirements [95]. Their use allows for precise control over dosage and timing, and they are often favored for short-term experiments or applications where regulated, transient expression is desirable.

In contrast, shRNAs are expressed intracellularly from DNA vectors, enabling long-term and stable gene knockdown due to continuous transcription [94]. This makes them particularly suitable for applications requiring prolonged silencing, such as *in vivo* disease models or stable cell lines. However, shRNA-based approaches involve nuclear transcription, export, and cytoplasmic processing by Dicer, introducing additional steps that may increase complexity and variability [97]. Moreover, high or unregulated expression of shRNAs—particularly when driven by strong promoters—has been associated with cytotoxicity [102], while their use can also lead to target-independent dysregulation of endogenous miRNAs and global transcriptomic changes [103]. The choice between siRNA and shRNA depends on the specific experimental objectives, the required duration and reversibility of gene silencing, and the biological context in which the RNAi is applied. Careful consideration of these factors is essential during both experimental design and therapeutic development.

**Table 1:** Comparative overview of siRNA and shRNA, two widely used molecules for RNAi-mediated RNA degradation. This table summarizes the key characteristics of small interfering RNA (siRNA) and short hairpin RNA (shRNA), highlighting their respective advantages and limitations in research and therapeutics.

Feature	siRNA	shRNA
Generation	Synthetic RNA duplexes delivered into cytoplasm [95,104]	Vector-expressed hairpin RNA transcribed in the nucleus [97,104]
Mechanism of Action	Direct incorporation into RISC; AGO2-mediated mRNA cleavage [95]	Transcription, export from nucleus, processed by Dicer into siRNA duplex, RISC loading [98]
Duration of Effect	Transient and rapid [104,105]	Long-lasting and stable due to continuous intracellular expression [97]
Delivery	Requires delivery vehicles (e.g., lipid nanoparticles) [106]	Delivered via plasmid or viral vectors [97,104,107]
Advantages	Precise control over dosage and timing; minimal processing [107]	Sustained knockdown; suitable for stable gene silencing [94,97]
Limitations	Susceptible to degradation; transient effect [104,108]	Risk of cytotoxicity; insertional mutagenesis; delayed onset [102,103]
Applications	Short-term gene silencing for *in vivo* functional studies [104,105]	*In vivo* disease models, stable cell line generation [108]

### 2.4. Emergence of CRISPR-Cas13 Systems

While RNAi represented a significant advancement in post-transcriptional gene regulation, the discovery of CRISPR-Cas system in the 2010s marked the next major leap in RNA manipulation technologies (Figure 1) [109,110]. Originally identified as an adaptive immune defense in bacteria [111], CRISPR-Cas systems were initially developed and adapted for genome editing applications [109,112,113,114,115]. However, the subsequent identification of Cas13 protein, an RNA-targeting effector, expanded the scope of CRISPR technologies beyond DNA editing [116,117,118]. Unlike Cas9, which induces double-stranded DNA breaks, Cas13 associates with a guide RNA (gRNA) that directs it to cleave single-stranded RNA (ssRNA) in a sequence-specific manner, without affecting genomic DNA [115,116]. This feature has enabled precise transcriptome modulation, facilitating functional RNA studies in cancer biology and laying the foundation for the development of novel RNA-targeting therapeutic strategies [119,120,121,122,123].

The Cas13 family is classified into 11 distinct subtypes: Cas13a, Cas13b, Cas13c, Cas13d, Cas13e, Cas13f, Cas13g, Cas13h, Cas13i, Cas13x, and Cas13y [124,125,126,127,128]. While all Cas13 proteins function as RNA-guided RNases, they differ in size, domain architecture, gRNA requirements, and functional efficiency in eukaryotic systems. Among the diverse Cas13 subtypes, Cas13a, Cas13b, and Cas13d have been most extensively characterized and optimized for use in mammalian cells, due to their robust activity and adaptability for transcriptome engineering applications [129,130,131,132].

#### 2.4.1. Cas13-Mediated Mechanism: From Recognition to Cleavage

Cas13-mediated RNA cleavage proceeds through a multistep process involving gRNA recognition, engagement, and catalytic cleavage [116]. Once introduced into the cell, Cas13 forms a ribonucleoprotein complex with the gRNA, which recruits Cas13 to the target RNA (Figure 3) [133]. In contrast to RNAi, which relies on host components like Dicer and Argonaute, Cas13 functions as a self-sufficient RNA-guided endonuclease [100].

Upon base-pairing between the gRNA and the target ssRNA, Cas13 undergoes a conformational rearrangement that activates its two Higher Eukaryotes and Prokaryotes Nucleotide-binding (HEPN) domains [134]. These domains come into close proximity, forming an active catalytic center that cleaves the target RNA, typically at flexible, unstructured regions near the hybridization site [134,135]. Unlike AGO2-mediated RNAi, Cas13 does not require a specific sequence motif for cleavage, allowing broader target flexibility.

#### 2.4.2. Current Limitations of the CRISPR-Cas13 System

CRISPR-Cas13 offers several advantages over traditional RNA-targeting technologies, including high specificity and efficient editing. Moreover, this system is highly flexible, allowing for the design of gRNAs that target a broad range of RNA molecules, enabling precise post-transcriptional modulation.

Despite its promise, the CRISPR-Cas13 system also faces notable limitations. One of the major challenges is the potential collateral activity that results in off-target effects [136]. Specifically, upon recognition and cleavage of the intended RNA target, Cas13 undergoes a conformational change that activates its HEPN nuclease domains. This activation can result in non-specific cleavage of nearby, non-target RNAs, potentially disrupting normal cellular function and confounding experimental results [137,138]. Collateral activity differs from traditional off-target effects, which also have been reported in CRISPR-Cas13 systems. More precisely, off-target effects are independent of the presence of the intended on-target, whereas collateral activity is reported only upon recognition of on-target RNA that perfectly matches the gRNA [133]. Recent studies have shown that expressing Cas13 or gRNA alone can trigger off-target effects, intrinsic targeting of host RNAs, and cellular toxicity [139]. Furthermore, while efficient and cell-type-specific delivery of CRISPR-Cas13 components *in vivo* remains an area of active development and is still challenging for specific tissues [140], recent studies have demonstrated that it can be effectively achieved in various tissues using adeno-associated viruses (AAVs) and polymer-based systems [141,142]. Furthermore, although Cas13 does not require protospacer adjacent motifs (PAMs) for target recognition, some orthologs (Cas13a, Cas13b, Cas13c) recognize protospacer flanking sequences (PFSs) [143], a restriction that should be taken under consideration when designing gRNAs.

#### 2.4.3. Optimizing CRISPR-Cas13 Systems: Recent Advances in Cas13-Based RNA Cleavage

Importantly, Cas13 variants have recently been engineered to overcome collateral activity [144]. A dual-fluorescence reporter system in mammalian cells was used to screen over 200 engineered Cas13 variants to assess collateral effects and on-target efficiency. The Cas13d and Cas13X variants demonstrated robust on-target activity with significantly reduced collateral effects. These high-fidelity variants exhibited minimal transcriptome-wide off-target effects, did not induce detectable cytotoxicity or cell growth arrest, and maintained RNA knockdown efficacy comparable to that of the wild-type enzyme. Importantly, *in vivo* assessments using transgenic mouse models and adeno-associated virus (AAV)-mediated delivery confirmed the absence of measurable collateral RNA cleavage, highlighting their safety and functional precision [42]. These high-fidelity Cas13 variants represent a major step forward in the development of more precise RNA-targeting technologies, offering enhanced specificity and safety profiles for both research and potential therapeutic applications.

#### 2.4.4. Beyond Cleavage: Expanding the Potential of Cas13-Based Systems in RNA Biology

A highly versatile advancement of the CRISPR-Cas13 platform is the engineering of catalytically inactive Cas13, commonly known as dead Cas13 (dCas13) [116]. This variant is generated through point mutations in the enzyme’s HEPN domains, effectively deactivating its intrinsic ribonuclease activity while retaining its sequence-specific RNA-binding capacity [145]. As a result, dCas13 functions as a programmable RNA-binding scaffold, capable of being fused to diverse effector domains to modulate RNA fate and function in a sequence-specific manner [146].

One of the most powerful applications of dCas13 is RNA base editing, particularly adenosine-to-inosine (A-to-I) editing. Fusion of dCas13 to catalytically active deaminase domains, such as adenosine deaminase acting on RNA 2 (ADAR2), enables site-specific editing of endogenous RNA transcripts (Figure 3) [147,148,149]. This technology underpins systems such as REPAIR (RNA Editing for Programmable A-to-I Replacement) and RESCUE (RNA Editing for Specific C-to-U Exchange) [145,150], which can allow transient and reversible correction of pathogenic point mutations at the RNA level, without altering the genome. This capability holds significant potential for modeling cancer-associated mutations, investigating their functional impact, and correcting oncogenic variants, contributing to the development of innovative RNA-targeted therapeutic strategies [149]. In addition to base editing, dCas13 has been exploited for performing epitranscriptomic changes by fusing it to RNA-modifying enzymes such as methyltransferases or demethylases, enzymes that alter N^6^-methyladenosine (m^6^A) marks [151,152,153]. These engineered systems enable site-specific addition or removal of specific chemical groups to ribonucleotides, offering new avenues for investigating how m^6^A and other epitranscriptomic marks regulate RNA stability, translation efficiency, and degradation [154,155,156,157]. Given the growing recognition of epitranscriptomic dysregulation in cancer, dCas13-based tools provide a powerful platform to explore the functional impact of RNA modifications on tumorigenesis, cancer progression, and therapeutic resistance [154].

Beyond RNA editing, dCas13 has proven effective in post-transcriptional gene regulation. For example, when fused to initiation factors, dCas13 can selectively enhance the translation of target mRNAs by promoting translational initiation [158]. In contrast, when dCas13 binds to the target mRNA near its 5′-end, it acts as a physical roadblock for scanning ribosomes, resulting in translational repression without affecting mRNA stability (Figure 3) [159]. Similarly, fusion of dCas13 to RNA-binding splicing factors (e.g., hnRNPa1 domains) enables programmable modulation of alternative splicing patterns at specific pre-mRNA sites [158,160]. This approach is particularly valuable for investigating and modulating cancer-associated splice variants implicated in cancer development, progression, and therapeutic resistance [161,162,163].

Additionally, dCas13 is a powerful tool for real-time RNA imaging in live cells [164]. When fused to fluorescent proteins (e.g., dCas13-GFP), it enables sequence-specific visualization of RNA localization, trafficking, and dynamic expression patterns without influencing endogenous RNA integrity (Figure 3) [165]. This application provides critical insight into the real-time subcellular localization of oncogenic transcripts, RNA–protein interactions, and transcriptomic reprogramming during cancer progression, cellular stress responses, and treatment-induced transcriptional remodeling [165,166,167].

dCas13 is a versatile and precise tool for studying and manipulating RNA molecules in a sequence-specific manner. Its expanding applications—including epitranscriptomic editing, splicing modulation, and live-cell RNA imaging—have made it especially valuable in cancer research. These capabilities provide powerful means to investigate RNA functions, the impacts of its dysregulation and to develop targeted RNA-based strategies for both diagnostics and potential therapies.

### 2.5. Small Molecules as RNA-Targeting Tools in Cancer Research

Small molecules have traditionally served as the foundation of drug discovery, primarily targeting proteins to modulate their function [168,169]. However, recent advances in RNA structural biology, high-throughput technologies, and computational modeling have broadened their application to include RNA molecules as direct targets [8,170]. This expansion is particularly significant in cancer research, where small molecules offer promising strategies to modulate or degrade dysregulated RNA transcripts—including oncogenic mRNAs, lncRNAs, and miRNAs—that contribute to tumor progression and resistance to therapy [171,172,173]. In contrast to ASOs, siRNA/shRNA, and CRISPR-Cas13 systems that rely on Watson–Crick base pairing, small molecules interact with RNA via non-covalent forces including hydrogen bonding, π–π stacking, and hydrophobic interactions [174]. These interactions enable the recognition of complex and often transient higher-order RNA structural motifs—such as internal loops, bulges, apical loops, pseudoknots, and G-quadruplexes (G4s)—thereby offering a structurally driven strategy for modulating RNA function in cells [174,175]. One particularly attractive class of RNA structures in oncology is G4s, motifs that can form in the untranslated regions (UTRs) of mRNAs and influence their stability and translational efficiency. G4s have been identified in the 5′ UTRs of several oncogenic transcripts, including those encoding NRAS and KRAS, where they act as translational repressors [176]. Stabilization of these structures using small-molecule ligands has been shown to inhibit translation of the associated oncogenic transcripts, offering a strategy to suppress cancer drivers at the RNA level [177,178]. Several small-molecule G4 ligands have demonstrated the ability to selectively bind and stabilize these motifs, leading to the suppression of oncogene expression in cancer cells [178,179].

#### 2.5.1. Functional Mechanisms of Small-Molecule RNA Targeting

**Figure 4:**
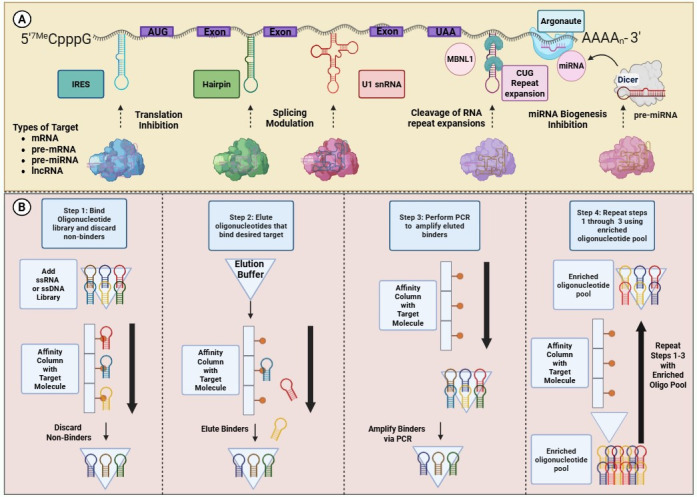
Small molecules and aptamers as tools for RNA targeting. (**A**) Schematic representation of the primary mechanisms by which small molecules target RNA. These compounds can modulate pre-mRNA splicing by inhibiting or stabilizing interactions with the splicing machinery, inhibit translation by binding to internal ribosome entry sites (IRES) and blocking ribosome binding, correct structural abnormalities (like expansions of CUG repeats) in mRNAs, and interfere with pre-miRNA disrupting its biogenesis. (**B**) Overview of the Systematic Evolution of Ligands by EXponential enrichment (SELEX) process, illustrating the iterative selection of aptamers with high affinity and specificity for a given RNA target.

RNA-targeting small molecules exert their therapeutic effects through diverse structure-dependent mechanisms, making them particularly promising in cancer treatment [174,180]. One major strategy involves allosteric inhibition of translation or ribonucleoprotein (RNP) complex formation. In this mechanism, small molecules bind to highly structured regions such as internal ribosome entry sites (IRES) or G4s within oncogenic mRNAs or viral mRNAs that are linked to cancer development, thereby blocking cap-independent translation and disrupting critical RNA–protein interactions required for mRNP assembly (Figure 4A) [181,182,183].

Another mechanism targets miRNA biogenesis, where compounds that bind to the stem-loop structures of primary or precursor miRNAs can interfere with processing by Drosha or Dicer [184], leading to decreased levels of oncogenic miRNAs in tumor cells [185,186]. Additionally, small molecules can modulate alternative splicing by stabilizing interactions between pre-mRNA and components of the spliceosome, such as U1 snRNP (Figure 4A) [187]. This mechanism can be utilized to promote exon inclusion and highlights the potential of splicing modulation in malignancies driven by aberrant splice variants [188].

Although classical small molecules do not typically induce RNA degradation through pathways like RNaseH1 or RISC, emerging approaches have adapted targeted degradation strategies for RNA. One such strategy is the development of ribonuclease-targeting chimeras (RiboTACs), which are bifunctional molecules consisting of an RNA-binding domain linked to a recruiter of endogenous ribonucleases like RNase L [189], that have been designed to selectively degrade RNAs associated with cancer [190,191]. More precisely, these chimeras can induce selective cleavage of oncogenic transcripts by leveraging proximity-induced enzymatic degradation [192], highlighting their significant potential in cancer treatment development. Compared to oligonucleotide-based therapeutics like ASOs and RNAi-based approaches, RIBOTACs are characterized by enhanced pharmacokinetic properties since—just like a small molecule—they are likely to have access to a broad range of tissues when administered to animals (Table 2) [193].

Finally, some small molecules function by stabilizing tumor-suppressive RNAs, including certain lncRNAs, enhancing their structural integrity and biological activity, and thus prolonging their half-life and tumor-suppressive functions within cancer cells [194]. These mechanisms underscore the versatility of small molecules as a platform for modulating RNA function in cancer research and therapeutics.

**Table 2:** Comparative summary of RNA-targeting approaches. This table provides a comparative overview of major RNA-targeting strategies applied in cancer research and therapeutics, including their mechanisms of action, primary molecular targets, advantages, and limitations.

Technique	Mechanism of Action	Primary Target	Advantages	Limitations
Gapmer ASOs [57,65,70]	Bind complementary RNA and recruit RNase H for RNA cleavage and degradation	mRNAs, lncRNAs	Enhanced binding affinity; nuclease resistance; reduced immunogenicity; high stability	Sequence- and structure-dependent hepatotoxicity and possible nephrotoxicity (driven by off-target RNase H1-mediated cleavage of pre-mRNAs); requires nuclear delivery; off-target effects; tumor heterogeneity;
SBOs [70,71,72]	Bind target RNA to sterically block translation or modulate splicing	Pre-mRNAs, mRNAs	Enables splicing or translation modulation without degrading RNA; lower risk of degradation-associated toxicity	Limited to steric interference; nuclear delivery still challenging; not suitable for complete gene knockdown; often requires nuclear delivery;
RNAi/ siRNAs [49,104]	Duplex siRNAs incorporated into RISC guide AGO2 to cleave perfectly matched RNAs	mRNAs	Potent knockdown; high sequence specificity; exploits endogenous machinery	Extracellular and intracellular barriers (e.g., nuclease degradation, poor membrane permeability, renal clearance, endosomal entrapment); risk of immune activation; off-target silencing; cytotoxicity;
RNAi/shRNAs [49,104,195]	Expressed shRNAs are processed by Dicer into siRNAs, loaded into RISC to degrade RNAs	mRNAs	Long-lasting effect via plasmid or viral delivery; uses endogenous RNAi machinery	Risk of insertional mutagenesis (viral vectors); potential competition with endogenous miRNA pathways; off-target effects; immune response;
AMOS [83,90]	Bind mature miRNAs to block interaction with endogenous mRNAs	Mature miRNAs	Specific inhibition of miRNA function	Possible off-target effects across related miRNA family members; challenges in delivery specificity and stability;
Aptamers [196,197,198]	Structured nucleic acid ligands bind RNA or other targets with high affinity	Structured RNA motifs	High specificity & affinity; low immunogenicity; customizable for diverse targets	Dependent on well-defined RNA structure; limited high-resolution structural data;
CRISPR–Cas13 Systems [50,112,119,129,133]	Cas13–gRNA complex binds and cleaves target ssRNA via HEPN domains	mRNAs, ncRNAs	Highly programmable, specific, reversible;	Challenging delivery to specific tissues; collateral cleavage can cause off-target RNA degradation and cytotoxicity; often requires PFS; immunogenicity concerns;
CRISPR–dCas13 Systems [158,159,160]	Catalytically inactive Cas13 binds RNA and directs fused effectors (editing, splicing)	mRNAs, pre-mRNAs, ncRNAs	Programmable modulation (splicing, editing, translation) without cleavage	Large size complicates delivery; immunogenicity; long-term safety and efficacy not yet fully evaluated; collateral effects remain a concern;
Small Molecules [51,168,170]	Bind RNA structural motifs to modulate interactions, splicing, translation, miRNA biogenesis	Structured RNA motifs	Chemically tunable; potential for diverse RNA function modulation	RNA structural variability; limited high-resolution structural data hinders rational design; risk of off-target binding

#### 2.5.2. Challenges in Targeting RNA with Small Molecules

Despite growing progress in targeting RNA with small molecules, significant barriers remain. One of the major challenges is the limited availability of high-resolution, experimentally validated RNA three-dimensional structures [180]. Since the efficacy of small molecules in modulating RNA function is dependent on recognizing specific secondary or tertiary structural motifs, a detailed understanding of the target RNA conformation is essential [174]. Unlike the extensive and diverse databases available for RNA sequences, current RNA structure repositories are disproportionately populated by a narrow subset of elements such as ribosomes and riboswitches [180], without significant structural data for cancer-associated RNAs, including lncRNAs, oncogenic mRNAs, and aberrantly spliced transcripts. This gap in knowledge impedes rational drug design and limits the application of structure-based modeling techniques.

Furthermore, despite major advances in protein structure prediction through deep learning algorithms—such as AlphaFold, which has revolutionized *de novo* protein modeling for drug discovery—analogous progress in RNA structural prediction remains restricted [199,200]. This gap stems primarily from the limited quantity and compositional diversity of experimentally determined RNA tertiary structures, which constrain the training of machine learning algorithms and impedes the generation of accurate, predictive models for RNA folding [201]. As a result, current computational tools lack the robustness required to reliably predict the complex and dynamic architectures of functional RNAs, particularly those implicated in cancer pathogenesis.

In addition, the chemical space for targeting RNA remains poorly investigated, as traditional drug design principles—optimized for proteins—do not apply to RNA [180]. Thus, designing compounds with appropriate physicochemical properties to selectively and potently bind structured RNA elements demands new, data-driven frameworks. Effective binding of a small molecule to the RNA target does not guarantee functional modulation, as high-affinity interactions may fail to induce the necessary conformational changes or disrupt critical RNA-mediated processes required for biological activity [180].

Another critical limitation in the field pertains to the development of heterobifunctional RNA-targeting compounds like RiboTACs [193]. While these molecules represent a promising strategy by harnessing endogenous ribonucleases to achieve selective RNA degradation, the current repertoire of effector modules (e.g., RNase recruiters) remains limited, thereby restricting the applicability of this platform across different RNA classes. Additionally, robust methods for target validation and transcriptome-wide selectivity profiling are not yet well-established. This lack of comprehensive profiling raises concerns about off-target effects and unintended interactions with structurally similar RNA elements, proteins, or even DNA [180].

Collectively, these limitations highlight the urgent need for integrative approaches that combine experimental RNA structural elucidation, advanced computational modeling, and transcriptome-wide functional screening to refine RNA-targeting strategies and enhance their therapeutic utility in cancer (Table 2).

### 2.6. Aptamers: Nucleic Acid Ligands for RNA Targeting in Oncology

Aptamers are synthetic single-stranded nucleic acids capable of folding into complex three-dimensional conformations that bind to protein targets with high specificity and affinity [202]. Aptamers with affinity for a desired target are selected from a large oligonucleotide library through a process called SELEX, which stands for Sequential Evolution of Ligands by Exponential Enrichment [203,204,205]. During this process, non-binding aptamers are discarded and aptamers binding to the proposed target are expanded. Initial positive selection rounds are sometimes followed by negative selection, a strategy that improves the selectivity of the resulting aptamer candidates. After multiple selection rounds, high-affinity aptamer candidates with optimal binding characteristics are isolated for downstream applications (Figure 4B) [203,206].

A recent breakthrough study has provided the first direct experimental evidence that aptamers can modulate RNA function by binding structured RNA targets without inducing degradation. Specifically, L-DNA aptamers were shown to recognize and bind with high affinity to precursor miR-155, a structured RNA critical in oncogenic pathways. Unlike traditional Watson–Crick base pairing used by antisense oligonucleotides or siRNAs, these L-DNA aptamers engage in cross-chiral interactions with D-RNA, demonstrating a unique mode of molecular recognition. Importantly, the bound aptamers effectively inhibited Dicer-mediated processing of pre-mir-155, thus blocking its maturation into the functional miRNA [207]. Notably, the sequences and binding modes of these L-DNA aptamers are distinct from previously reported L-RNA aptamers, suggesting a diverse landscape of aptamer–RNA recognition strategies. This study not only establishes the feasibility of direct RNA targeting by aptamers but also highlights the potential of L-DNA as a chemically robust and biologically stable platform for therapeutic applications. Despite this promise, further work is needed to validate the generalizability of this approach to other structured RNAs and to evaluate its efficacy *in vivo*.

Aptamers are also emerging as biosensors for RNA detection in diagnostic applications. Their high affinity, specificity, and ability to undergo conformational changes upon target binding make them ideal molecular recognition elements in biosensor platforms. Aptamer-based biosensors (aptasensors) have been developed for the sensitive and selective detection of oncogenic RNAs (Figure 2), including dysregulated miRNAs commonly associated with cancer [208,209]. These biosensors can be integrated with various signal transduction modalities, such as electrochemical, optical, or fluorescence-based systems, enabling real-time and label-free detection. Their chemical stability, ease of modification, and low immunogenicity offer key advantages over traditional antibody-based detection methods [210]. In oncology, aptasensors hold promise for early disease detection, monitoring of treatment response, and real-time quantification of therapeutic targets, thus providing a complementary diagnostic modality to aptamer-based therapeutics within an integrated precision medicine framework.

#### Exploiting Aptamers for the Generation of Aptamer-Oligonucleotide Chimeras in RNA Therapeutics

Their programmable nature, small size, and low immunogenicity make aptamers particularly attractive not only for targeting RNA molecules directly but also for delivering RNA-based therapeutics to specific cell types. In recent years, aptamer-oligonucleotide chimeras have emerged as a powerful strategy to enhance the precision, efficacy, and safety of RNA-based therapeutics. [198,211]. In detail, aptamers can be chemically or enzymatically fused to therapeutic oligonucleotides, forming aptamer-oligonucleotide chimeras that combine the cell-targeting capabilities of aptamers with the previously described functions of siRNAs, shRNAs, AMOs, or ASOs [196,197,212,213,214].

Aptamer-siRNA chimeras (AsiCs) enable receptor-specific gene silencing and have been evaluated in preclinical models for cancer therapy and immune modulation. For instance, an EpCAM-targeting aptamer fused to a siRNA against Polo-like kinase 1 (*PLK1*) effectively reduced tumor burden in breast cancer models [215]. Modifications such as the addition of 3′ di-uridine overhangs to enhance DICER recognition, or incorporation of guide strand–aptamer fusions with optimized stem-loop structures, have been shown to improve processing and silencing efficiency [215,216,217]. Furthermore, AsiCs have been engineered to sensitize immune-refractory tumors to checkpoint blockade therapy, as demonstrated by a chimera consisting of the nucleolin-targeting aptamer AS1411 conjugated to a siRNA against *SMG1*. This construct enhanced tumor antigenicity and promoted responsiveness to immunotherapy without inducing immune activation in healthy tissues [218].

Furthermore, shRNAs can offer prolonged gene silencing by mimicking endogenous precursor miRNAs and being transcribed from DNA templates [107,196]. Aptamer-shRNA chimeras can be synthesized as single molecules using PCR-based or co-transcriptional approaches, improving delivery efficiency and simplifying production workflows [219,220]. Therapeutic efficacy has been demonstrated in models targeting *RORGT* in Th17 cells, where a CD30-targeting aptamer-shRNA chimera effectively silenced *RORGT* and reduced inflammatory cytokine expression [219], as well as in prostate cancer, where a PSMA-targeting aptamer-shRNA against *DNA-PK* enhanced tumor radiosensitivity and extended survival in preclinical models [196]. These findings highlight the promising potential of aptamer-shRNA constructs in autoimmune and cancer therapies.

Aptamer-anti-miRNA chimeras are constructs that enable cell-specific inhibition of oncogenic miRNAs, offering a targeted approach to modulate dysregulated miRNA pathways in disease. A notable example is GL21.T aptamer, which targets the AXL receptor tyrosine kinase and is conjugated to an anti-miR-222. This chimera (GL21.T–anti-miR-222) demonstrated therapeutic efficacy in glioblastoma models by reducing miR-222 levels, suppressing AXL activity, and significantly inhibiting tumor growth following intravenous administration [213]. Both linear and bivalent designs have been investigated to improve silencing efficiency and enable multitargeting capabilities, such as incorporating multiple anti-miRNAs within a single construct [196,213]. These strategies enhance therapeutic flexibility and broaden the potential application of aptamer–anti-miRNAs chimeras across different miRNA-driven cancer types.

Finally, aptamer-ASO chimeras provide a targeted strategy to inhibit specific transcripts with enhanced specificity and stability. For example, a PD-L1-targeting aptamer-ASO chimera effectively suppressed *PD-L1* expression in a murine colorectal cancer model, reducing tumor burden to levels comparable to anti-PD-L1 antibody therapy. Importantly, this construct also decreased levels of soluble PD-L1, overcoming key mechanisms of immune evasion [212]. Additionally, circularized aptamer-ASO constructs, generated by ligating the 5′ end of the aptamer to the 3′ end of the ASO, demonstrated improved serum stability and durable silencing activity. These circular chimeras exhibited potent inhibition of SARS-CoV-2 N gene transcripts *in vitro*, highlighting their therapeutic potential in infectious disease contexts beyond oncology [221].

## 3. The Significance of RNA-Targeting Techniques in Cancer Research and Therapy

RNA-targeting strategies have emerged as powerful and versatile tools in cancer research and therapy, offering novel insights into the molecular mechanisms underlying tumor development, progression, and drug resistance [222,223,224,225]. The recognition that RNA molecules play critical regulatory roles in a wide array of biological processes has markedly expanded their potential as therapeutic targets, while advances in RNA biology, high-throughput sequencing technologies, and transcriptomic profiling have further transformed our understanding of cancer pathogenesis and paved the way for innovative therapeutic interventions [77,226,227]. Unlike traditional approaches that primarily target proteins, RNA-targeting techniques enable the direct and selective modulation of gene expression at the post-transcriptional level, offering the ability to intervene more precisely in disease pathways.

One of the most promising contributions of RNA-targeting strategies in oncology is the ability to degrade or inhibit specific mRNAs that encode oncogenic proteins, thereby mitigating the disease phenotype. ASOs, CRISPR-Cas13 systems, and siRNAs have shown efficacy in silencing mRNAs involved in key cancer-related pathways, such as those regulating cell proliferation, apoptosis, and angiogenesis [223,228,229]. For example, RNA-based approaches that target mutant *KRAS* mRNA—commonly implicated in pancreatic, lung, and colorectal cancers—are being explored as potential therapeutics to inhibit one of the most elusive oncogenic drivers in solid tumors [228,230,231]. By reducing the production of pathogenic proteins directly at the RNA level, these therapies can offer enhanced specificity and reduced off-target toxicity compared to traditional small-molecule inhibitors that target proteins [232].

Beyond targeting mRNAs, RNA-targeting techniques have also advanced our ability to study and therapeutically modulate ncRNAs, which are increasingly recognized as key regulators of cancer biology [233,234,235,236]. For instance, miRNAs can act as oncogenic or tumor suppressor molecules by regulating gene expression post-transcriptionally. Dysregulation of miRNA expression profiles is a hallmark of many cancers, contributing to uncontrolled cell growth, metastasis, and resistance to therapy [237,238]. Therapeutic strategies involving miRNA inhibitors like AMOs or miRNA sponges are being developed to restore normal regulatory function and suppress tumor progression [239,240]. Similarly, lncRNAs and circRNAs, which control chromatin architecture, transcription, and signaling networks, are emerging as novel targets for RNA-based therapeutics in cancer [228,241].

Small molecules and aptamers are expanding the RNA-targeting toolkit, as they are increasingly being recognized as powerful RNA-targeting modalities in oncology. Small molecules offer unique advantages by modulating RNA structure and function, enabling the selective targeting of complex RNA motifs such as G4s, IRES elements, and splice sites involved in cancer pathogenesis [176,180,181,188]. Emerging platforms like RiboTACs extend this capability by coupling RNA binding to catalytic RNA degradation, providing enhanced therapeutic precision and bioavailability [193]. Aptamers, by contrast, act as highly specific nucleic acid ligands that can bind RNA targets or deliver RNA therapeutics directly to cancer cells via aptamer-oligonucleotide chimeras [196,197,212,215]. Their ability to block miRNA processing or serve as tumor-selective delivery vehicles significantly expands the utility of RNA-targeting approaches for both therapeutic and diagnostic applications [197,208].

Importantly, RNA-targeting approaches also offer promise in overcoming drug resistance—one major challenge in cancer therapy. By selectively silencing resistance-associated genes or modifying splicing patterns that give rise to resistant isoforms, RNA-based approaches can enhance the efficacy of existing treatments and prevent relapse [242,243]. Additionally, RNA-editing tools and ssASOs provide a means to correct aberrant splicing events commonly seen in cancer, such as those affecting tumor suppressor genes like *TP53* or *BRCA2* [242,244,245].

As cancer research continues to evolve, RNA-targeting techniques are emerging as a critical modality in the development of next-generation molecular therapies. By enabling precise modulation of both coding and non-coding RNAs involved in tumorigenesis, these approaches offer innovative ways to overcome the limitations of traditional treatments, facilitating the advancement of personalized therapeutic strategies, and potentially improving clinical outcomes in cancer patients.

## 4. Current Landscape of FDA-Approved RNA-Targeting Therapies: A Focus on Cancer

RNA-targeting therapies have emerged as transformative tools in modern medicine, particularly in the treatment of genetic disorders [246,247,248]. By manipulating RNA molecules directly, these therapies enable precise modulation of gene expression either by altering pre-mRNA splicing, degrading oncogenic mRNAs, or inhibiting the translation of disease-causing transcripts (Figure 2) [74,249,250]. ASOs and siRNA-based approaches have been introduced as leading RNA-targeting modalities, with several agents achieving FDA approval—primarily for non-cancerous conditions.

Notable examples include nusinersen (Spinraza), a ssASO approved in 2016 for the treatment of spinal muscular atrophy (SMA), which corrects *SMN2* splicing to increase the production of the survival motor neuron (SMN) protein [251,252]. Similarly, eteplirsen, approved for Duchenne muscular dystrophy, induces exon skipping in the dystrophin transcript to partially restore protein function [253,254]. In siRNA-based therapeutics, patisiran (Onpattro) became the first FDA-approved siRNA drug in 2018, targeting *TTR* mRNA to treat hereditary transthyretin-mediated amyloidosis (hATTR) by reducing toxic protein accumulation [195,248]. Additionally, risdiplam (Evrysdi), approved in 2020 for SMA, became the first non-ribosomal small molecule that was shown to directly modulate RNA splicing [255]. It binds to the *SMN2* pre-mRNA, enhancing exon 7 inclusion and restoring SMN protein levels [256]. Together, these agents highlight the therapeutic versatility of RNA-targeting approaches across a range of genetic disorders and have paved the way for their application in more complex diseases like cancer.

Despite these successes, the utilization of RNA-targeted therapies in oncology has faced significant challenges. Unlike the described disorders, which typically involve a single, well-defined genetic defect, cancer is a multifaceted and highly heterogeneous disease, often driven by a complex interplay of multiple genetic and epigenetic factors [257,258]. A major challenge in RNA-targeted cancer therapy is the identification of RNA targets that are indispensable for tumor cell survival while exhibiting high specificity to minimize off-target effects on normal cells. Furthermore, the inherent genetic heterogeneity and rapid evolution of cancer cells frequently diminish the durability of treatments aimed at a single target, necessitating strategies that can address tumor adaptability and resistance.

Until recently, no RNA-targeting therapy had been approved for cancer, despite extensive preclinical efforts involving ASOs, siRNAs, CRISPR-Cas13, aptamers, and RNA-modulating small molecules. However, in mid-2024, imetelstat became the first FDA-approved RNA-targeting drug for cancer indication (Figure 1 and Table 3). Imetelstat is an ASO-based telomerase inhibitor that binds to the RNA component of the enzyme (*TERC*) and was approved for the treatment of lower-risk myelodysplastic syndromes [259,260]. This milestone not only validates the clinical potential of RNA-targeting strategies in oncology but also underscores the feasibility of targeting ncRNAs involved in essential cancer pathways.

Although imetelstat currently stands as the only FDA-approved RNA-targeting therapy for cancer, numerous other RNA-targeting agents are undergoing clinical evaluation. These ongoing efforts highlight the therapeutic potential of these approaches in cancer treatment and disease management, while expanding the scope of precision medicine by enabling the modulation of previously inaccessible targets.

## 5. Targeting RNA in Cancer: Insights from Current Clinical Trials

The application of RNA-targeting technologies in oncology is advancing rapidly, driven by their ability to modulate gene expression at the transcriptomic level with high specificity and functional precision. Various therapeutic platforms—including ASOs and siRNAs—are currently being evaluated in clinical trials for diverse cancer types [261,262]. These approaches are designed to selectively degrade oncogenic transcripts, modulate alternative splicing events, or disrupt pathological RNA–protein interactions (Figure 2), thereby targeting key mechanisms underlying cancer cell survival, proliferation, and metastasis.

### 5.1. Advancing ASOs in Human Clinical Trials

Although ASO-based therapies have not yet achieved broad regulatory approval in oncology, several candidates have advanced to mid- and late-stage clinical trials, demonstrating growing interest in their therapeutic potential. One prominent example is danvatirsen (AZD9150), a next-generation ASO that targets *STAT3* mRNA, a transcription factor implicated in tumor growth and immune evasion [263]. Danvatirsen is currently being investigated in an ongoing Phase II clinical trial (NCT02983578) in combination with the PDL1 inhibitor durvalumab, in patients with non-small cell lung cancer (NSCLC), advanced pancreatic cancer, and mismatch repair-deficient (dMMR) colorectal cancer (Table 3). While Phase I trials have indicated favorable safety and preliminary antitumor activity, definitive efficacy data are pending, and the therapy remains in the investigational stage [264]. Another candidate, BP1002, is an ASO targeting *BCL2* mRNA, a key anti-apoptotic factor frequently overexpressed in hematologic malignancies. BP1002 is currently under evaluation in a Phase I clinical trial (NCT04072458) for patients with relapsed or refractory lymphoid malignancies [265]. Additional ASO candidates, including OT-101 (targeting *TGFB2* mRNA), WGI-0301 (targeting *AKT1* mRNA), and BP1001 (*GRB2*), are currently undergoing early-phase clinical evaluation in solid tumors [266,267,268]. These efforts highlight the versatility of ASOs in targeting coding RNAs implicated in oncogenesis, even as challenges related to delivery, specificity, and durability of response remain under investigation.

### 5.2. siRNA-Based Therapies in Clinical Trials

Although no siRNA therapy has received FDA approval for cancer treatment thus far, multiple siRNA candidates targeting oncogenic transcripts have advanced into clinical trials, underscoring the therapeutic potential of RNAi in oncology. A promising agent is EPHA2-targeting siRNA, developed for treating patients with advanced solid tumors that often exhibit resistance to standard therapies. This siRNA functions by specifically silencing *EPHA2* mRNA, a receptor tyrosine kinase overexpressed in many cancers and associated with tumor growth, angiogenesis, and metastasis [269]. This agent is currently being evaluated in a Phase I clinical trial to assess its safety profile and determine the optimal dosing regimen [270]. A novel siRNA therapeutic targeting NUDT21 (NCT06424301) has been developed for the treatment of retinoblastoma (RB) [271]. While current approaches such as intra-arterial and intravitreal chemotherapy have improved outcomes, they remain limited by toxicity and drug resistance [272]. This siRNA selectively silences *NUDT21*, a regulator of 3′UTR processing that promotes tumor proliferation through *SMC1A* stabilization. Preclinical studies have shown that *NUDT21* inhibition suppresses tumor growth with minimal off-target toxicity. Delivered via intraocular injection, this agent represents a first-in-class approach for RB and is now being evaluated in an early-phase clinical trial to assess safety and preliminary efficacy in refractory cases [271]. Additionally, STP705, a first-in-class siRNA therapeutic simultaneously targeting *TGFB1* and *COX2* mRNAs, has shown remarkable efficacy in a Phase II trial (NCT04669808) for cutaneous basal cell carcinoma, achieving a 100% complete response rate with an excellent safety profile (Table 3) [273]. This dual-targeting approach inhibits two critical drivers of tumor progression and inflammation, offering a novel therapeutic avenue in oncology.

### 5.3. CRISPR-Cas13-Based Approaches: Still in Preclinical Stage for Cancer

Although CRISPR-Cas13 systems represent a relatively recent advance in RNA-targeting technology—first characterized less than a decade ago—they have already entered clinical trials for non-cancer indications, highlighting their exceptional translational potential. While no Cas13-based therapies for cancer treatment have yet been tested in humans, early-phase clinical programs in non-oncologic diseases have demonstrated both feasibility and safety. Notably, HuidaGene Therapeutics has launched two first-in-human Cas13 programs: the HERO trial, evaluating HG204 for *MECP2* Duplication Syndrome (MDS), and the SIGHT-I and BRIGHT trials, testing HG202 for neovascular age-related macular degeneration (nAMD). These therapies harness Cas13′s ability to selectively degrade pathogenic RNAs—*MECP2* mRNA in MDS and *VEGFA* mRNA in nAMD—offering a programmable, transcript-specific approach for treating these diseases [274,275]. Importantly, HG202 received FDA clearance in 2024, becoming the first Cas13-based RNA-editing therapy authorized for human testing in the USA [275].

Although still in preclinical stages for oncology, several studies have already demonstrated the potential of Cas13 systems in cancer models. For instance, in glioma models, lentiviral overexpression of LwCas13a induced a collateral RNA cleavage effect upon targeting EGFP and the tumor-specific EGFRvIII mutant, resulting in selective tumor cell death and inhibition of intracranial tumor growth. Bulk and single-cell RNA sequencing confirmed this effect, highlighting Cas13a’s tumor-eliminating capability [276]. In bladder cancer, a targeted liposome delivery system transported Cas13a gene circuits controlled by tumor-specific promoters and activated by near-infrared light. This system enabled multiplexed crRNA targeting and receptor-specific delivery without genomic disruption, offering a versatile gene therapy approach [277]. Furthermore, to overcome challenges in spatial and temporal regulation of Cas13a activity, innovative delivery systems such as hierarchical RNA nanococoons (RNCOs-D) have been developed. These nanococoons encapsulate Cas13a ribonucleoproteins along with chemotherapeutic agents and remain inactive until exposed to the acidic tumor microenvironment, which triggers their disassembly and reactivates Cas13a. This activation is further refined by the specific cleavage of *EGFRvIII* mRNA, enabling targeted silencing of this oncogenic transcript. highlighting a promising strategy for precise, controlled, and multimodal cancer therapy using CRISPR-Cas13a [278].

These milestones strongly support the clinical viability of Cas13 technologies and pave the way for future cancer applications. With ongoing progress in delivery systems and tumor-selective targeting, CRISPR-Cas13 holds a significant promise to overcome current therapeutic limitations in cancer, offering precise, reversible control over oncogenic transcripts.

### 5.4. Small Molecules and Aptamers Targeting RNA: Current Landscape and Prospects

Despite growing interest in RNA as a therapeutic target, there are currently no small-molecule drugs in clinical trials for cancer that directly bind and target RNA molecules. While several small molecules have been investigated for their ability to modulate RNA-related processes—such as H3B-8800 and E7107—these agents act by targeting proteins involved in RNA processing, rather than RNA itself [279,280,281]. Similarly, PRMT5 inhibitors, such as JNJ-64619178 currently in Phase I clinical trials for cancer (NCT03573310), modulate RNA splicing by altering the methylation status of splicing-related proteins rather than through direct RNA binding [282]. Although these compounds influence RNA biology and transcriptome dynamics, their mechanisms are indirect, relying on disruption of RNA-associated proteins rather than structural or sequence-specific interaction with RNA. Similarly, while several aptamer therapeutics have entered clinical trials for cancer, none function by binding RNA molecules directly to exert their anticancer effects. A notable agent, NOX-A12 (olaptesed pegol), is a Spiegelmer L-RNA aptamer that binds the chemokine CXCL12 [283], modulating the tumor microenvironment. Currently undergoing Phase I/II trials in glioblastoma (NCT04121455) [284], NOX-A12 highlights the clinical potential of aptamers in cancer therapeutics. To date, no aptamer in active clinical evaluation in oncology binds directly to RNA targets such as oncogenic mRNAs or structured ncRNAs, since the study of these molecules remain restricted to preclinical research.

Nonetheless, recent advances in RNA structural biology, high-throughput screening, and computational drug design are now enabling the development of small molecules and aptamers that directly bind RNA with increasing specificity and affinity. These efforts are leading to the identification of preclinical candidates that bind defined RNA motifs, offering a promising new direction for transcriptome-targeted cancer therapeutics.

**Table 3:** Clinical status of RNA-targeting therapies for cancer. This table summarizes key RNA-targeting therapeutic agents and their current status in clinical settings. Non-cancer indications are included for comparison, emphasizing the translational potential of RNA-targeting modalities.

Therapeutic Agent	Target	RNA-Targeting Strategy	Clinical Status	FDA Approval & Indication
Imetelstat (Rytelo)	*TERC*	ASO	FDA-approved (2024)	Approved for adult patients with low- to intermediate-1 risk myelodysplastic syndromes (MDS) [259,260].
danvatirsen (AZD9150)	*STAT3* mRNA	ASO	Phase II clinical trial	Investigated in combination with durvalumab for NSCLC, advanced pancreatic cancer, and dMMR colorectal cancer; safety promising, efficacy pending [264].
BP1002	*BCL2* mRNA	ASO	Phase I clinical trial	Evaluated in patients with relapsed or refractory lymphoid malignancies [265].
OT-101	*TGFB2* mRNA	ASO	Early-phase clinical trials	Under evaluation in various solid tumor types [268].
WGI-0301	*AKT1* mRNA	ASO	Early-phase clinical trials	Under evaluation [267].
BP1001	*GRB2* mRNA	ASO	Early-phase clinical trials	Under evaluation [266].
EPHA2-targeting siRNA	*EPHA2* mRNA	RNAi/siRNA	Phase I clinical trial	Targeting *EPHA2* mRNA in advanced solid tumors; safety profile and dosing under assessment [270].
NUDT21-targeting siRNA	*NUDT21* mRNA	RNAi/siRNA	Early-phase clinical trial	First-in-class siRNA for retinoblastoma; delivered intraocularly; safety and preliminary efficacy under evaluation [271].
STP705 (dual-target siRNA)	*TGFΒ1* & *COX2* mRNAs	RNAi/siRNA	Phase II clinical trial	Achieved 100% complete response in cutaneous basal cell carcinoma with excellent safety profile [273].
HG202	*VEGFA* mRNA (non-cancer)	CRISPR-Cas13	Early human clinical trials	FDA-cleared for clinical trials in neovascular age-related macular degeneration (nAMD); first Cas13-based RNA-editing therapy in human testing [275].

## 6. RNA-Based Therapeutics in Cancer: Current Advances and Future Perspectives

RNA-targeting strategies are at the forefront of molecular oncology, marking a significant advancement in how drugs are developed to treat cancer. While no siRNA or Cas13-based therapies have yet received FDA approval for cancer indications, the growing number of clinical trials highlights increasing confidence in RNA molecules as effective therapeutic targets. A major milestone was achieved in 2024 with the approval of imetelstat, marking the first FDA-approved ASO therapy for cancer and demonstrating regulatory acceptance of RNA-based approaches in cancer therapy [260]. Numerous other ASO and siRNA candidates are currently in Phase I/II trials, addressing hematologic and solid malignancies with encouraging preliminary results.

At the same time, the RNA-targeting field is moving beyond traditional approaches. CRISPR-Cas13 systems, while still in preclinical development for cancer [112,116,145,146], have reached clinical testing in non-cancer diseases, laying the groundwork for future oncology applications [285]. Small molecules that directly bind RNA, although not yet entered clinical trials for cancer, are increasingly identified from high-throughput screens and structure-guided design efforts. These efforts focus on targeting well-defined structural motifs within non-coding RNAs and oncogenic mRNA that play critical roles in cancer. Additionally, advances in delivery systems are helping to overcome critical challenges in RNA stability, biodistribution, and intracellular uptake [286].

Looking forward, future research will likely focus on combining therapies that use RNA-targeting agents alongside immunotherapies, kinase inhibitors, or chemotherapies to overcome treatment resistance and enhance the longevity of patient responses. Personalized RNA-targeting approaches, guided by detailed analysis of tumor transcriptomes, alternative splicing patterns, and RNA modifications, are expected to play an increasingly important role in cancer treatment. As this field advances, RNA-targeting therapies have the potential to transform oncology by enabling precise, versatile, and tumor-specific molecular interventions.

## 7. Conclusions

RNA-targeting strategies are rapidly emerging as powerful tools in cancer research and therapy, enabling precise modulation of gene expression at the RNA level. Unlike traditional treatments that act on proteins, these approaches can selectively silence oncogenic transcripts or correct aberrant splicing, offering high specificity with reduced toxicity. The recent approval of imetelstat, which targets the RNA component of telomerase, marks a major milestone and validates the clinical potential of RNA-targeting strategies in oncology. Advances in RNA biology, high-throughput sequencing techniques, and transcriptome profiling are ushering in a new era of innovation in the field. Future efforts will likely focus on combining RNA-targeted therapies with existing cancer treatments and developing personalized approaches based on tumor-specific RNA signatures. Overall, RNA-based therapeutics represent a versatile and promising approach to overcoming the complexity and resistance mechanisms inherent in cancer.

This review offers novel contributions compared to existing literature:

- Comprehensive comparative analysis of key RNA-targeting platforms, including antisense oligonucleotides, RNA interference, CRISPR-Cas13 systems, and RNA-binding small molecules, is performed, emphasizing their distinct mechanisms and applications in oncology.
- Discussion of recent clinical and preclinical advancements is presented with an emphasis on translational relevance, highlighting how RNA-targeting approaches are progressing toward real-world cancer treatments.
- A forward-looking perspective that outlines future research directions, including personalized RNA therapeutics based on tumor-specific transcriptomic signatures, is presented.

## Data Availability

No new data were created or analyzed in this study.
